# The relationship of plasma *Trans* fatty acids with dietary inflammatory index among US adults

**DOI:** 10.1186/s12944-017-0527-1

**Published:** 2017-08-04

**Authors:** Mohsen Mazidi, Hong-kai Gao, Nitin Shivappa, Michael D. Wirth, James R Hebert, Andre Pascal Kengne

**Affiliations:** 1 Key State Laboratory of Molecular Developmental Biology, Institute of Genetics and Developmental Biology, Chinese Academy of Sciences, Beijing, 100101 China; 2 Institute of Genetics and Developmental Biology, International College, the University of Chinese Academy of Science, Beijing, 100101 China; 3 The General Hospital of Chinese People’s Armed Police Forces, Beijing, China; 4 Cancer Prevention and Control Program, University of South Carolina, 915 Greene Street, Suite 200, Columbia, SC 29208 USA; 5 Department of Epidemiology and Biostatistics, University of South Carolina, 915 Greene Street, Suite 400, Columbia, SC 29208 USA; 6 Connecting Health Innovations, LLC, 1417 Gregg Street, Columbia, SC 29201 USA; 7 Non-Communicable Diseases Research Unit, South African Medical Research Council and University of Cape Town, Cape Town, South Africa

**Keywords:** *Trans* fatty acids, Dietary inflammatory index, National Health and nutrition examination survey

## Abstract

**Background:**

It has been suggested that *trans* fatty acids (TFAs) play an important role in cardiovascular diseases. We investigated the association between plasma TFAs and the dietary inflammatory index (DII) ™ in US adults.

**Methods:**

National Health and Nutrition Examination Survey (NHANES) participants with data on plasma TFAs measured from 1999 to 2010 were included. Energy-adjusted-DII ™ (E-DII ™) expressed per 1000 kcal was calculated from 24-h dietary recalls. All statistical analyses accounted for the survey design and sample weights.

**Results:**

Of the 5446 eligible participants, 46.8% (*n* = 2550) were men. The mean age of the population was 47.1 years overall, 47.8 years for men and 46.5 years for women (*p* = 0.09). After adjustment for C-reactive protein, body-mass-index, smoking, race, age, education, and marital status in linear regressions, *trans* 9-hexadecenoic acid [β coefficient 0.068 (95% CI: 0.032 to 0.188)], *trans* 11-octadecenoic acid [β coefficient 0.143 (95% CI: 0.155 to 0.310)], *trans* 9-octadecenoic acid [β coefficient 0.122 (95% CI: 0.120 to 0.277)], *trans* 9, and *trans* 12-octadienoic acid [β coefficient 0.103 (95% CI: 0.090 to 0.247)] were positively associated with the DII (all *p* < 0.001).

**Conclusion:**

The association of plasma TFAs with a marker of dietary inflammation suggests an underlying mechanism in the initiation and progression of cardiovascular diseases.

## Background

*Trans*-fatty acids (TFAs) are unsaturated fatty acids with at least one unsaturated, non-conjugated double bond in the *trans* (rather than the typical *cis*) configuration. Because humans do not synthesize trans-fatty acids, levels of these fatty acid isomers in serum depend on dietary intake. TFAs occur naturally in fat from ruminant animal meat, milk, and dairy fat and artificially in industrially hardened vegetable oils [1]. Dietary exposure to partially hydrogenated vegetable oils occurs through consumption of margarine and industrially processed foods such as cakes, candies, cookies, chocolate, mayonnaise, potato chips, French fries and other deep-fat fried foods, and fast food more generally [2].

Increased dietary intake of TFAs is linked with incident cardiovascular disease (CVD), and Type 2 diabetes mellitus (T2DM) [3–6]. A recent observational study [7] and a short-term randomized trial [8] have indicated that TFAs intake increases systemic inflammation in generally healthy individuals. Because systemic inflammation is an independent risk factor for future CVD [9], these findings suggest a potential mechanism whereby TFAs may affect cardiovascular health. Recommendations from the American Heart Association have led 13 local governments to implement a TFA ban [10] and the Food and Drug Administration to require the TFAs content to be listed on the nutrition facts panel of foods and dietary supplements [10].

Pro- or anti-inflammatory properties have been described for a number of foods and nutrients, and extensive literature research on these food items and nutrients has led to the development of the Dietary Inflammatory Index (DII)™ [11]. The goal of this index was to use different food parameters, categorized as pro- or anti-inflammatory, in order to calculate the inflammatory property of a person’s total diet [11, 12]. Previously, the DII was associated with a range of outcomes including markers of systemic inflammation, CVD, bone mineral density, telomere length, and overall mortality [13–15]. A relationship between the DII and levels of serum inflammatory markers has been found in multiple cohorts [11, 13, 16, 17]. However, no study has evaluated the association between plasma TFA concentration and DII scores. Therefore, our aim was to investigate the association between plasma TFA as an indicator of inflammation and the DII in US adults. We hypothesized that concentration of TFAs in serum will be higher with increasing DII scores.

## Methods

### Population

The National Health and Nutrition Examination Surveys (NHANES) are ongoing repeated cross sectional studies performed by the US National Center for Health Statistics (NCHS) [18]. NHANES applies a multistage probability sampling strategy, that oversamples certain subgroups of the U.S. population [19]. The NCHS Research Ethics Review Board approved the NHANES protocol and consent was obtained from all participants. Approximately 5000 subjects participate in NHANES each year, and the data are stated in 2-year cycles accessible for public domain [20]. Data collection on demographic, dietary, and behavioral information occurs through in-home administered questionnaires, though anthropometric measurements and biomarker variables are collected by trained subjects applying the mobile exam clinics (MEC). The interview consists of questions on socio-demographic characteristics (age, gender, education and race/Hispanic origin). More comprehensive information on the NHANES protocol is accessible elsewhere [21]. This study was based on analysis of data from the 1999 to 2010 NHANES cycles. Analyses were limited to subjects aged 18 years and older.

### Plasma trans fatty acids

This measurement procedure determines the total (free and esterified) content of selected *TFAs* in plasma and provides results in concentration units as well as percent units (*trans* fatty acids as percent of total fatty acids). The fatty acids in plasma are converted into free fatty acids by subsequent acidic and alkaline hydrolysis. The free fatty acids are extracted from the sample solution using liquid-liquid extraction and derivatized with pentafluorobenzylbromide (PFB-Br). The derivatized fatty acids are separated by capillary gas chromatography and detected by mass spectrometry using negative chemical ionization. The fatty acids are identified based on their chromatographic retention time and on the specific mass to charge ratio of the ion formed in the ion source. Retention times are compared against those obtained with known standards. Quantitation is performed with standard solution using stable isotope-labelled fatty acids as internal standards. To calculate *trans* fatty acids as percent of total fatty acids, 29 fatty acids are determined with this measurement procedure. These fatty acids cover over 95% of all fatty acids reported in plasma. This method determines the following four *trans* fatty acids: *trans*-9-hexadecenoic acid (palmitelaidic acid, C16:1n-7 t), *trans*-9-octadecenoic acid (elaidic acid, C18:1n-9 t), *trans*-11-octadecenoic acid (vaccenic acid, C18:1n-7 t-), *trans*-9, trans-12-octadecadienoic acid (linolelaidic acid, C18:2n-6 t, 9 t) [22].

### Diet and dietary inflammatory index

The development and validation of the DII have been described in detail elsewhere [12]. In short, nearly 2000 research articles, published between 1950 and 2010, examining the relationship between components of diet and pro-inflammatory cytokines (i.e., IL-1β, IL-6, tumour necrosis factor [TNF]-α, and C-reactive protein (CRP)) and anti-inflammatory cytokines (IL-4 and IL-10) were reviewed. This search revealed that 45 different food parameters (mostly micro, macro nutrients and flavanoids plus some individual food items) had a sufficiently robust literature to allow for scoring. Articles showing a positive association between the food parameters and the pro-inflammatory cytokines or a negative association with anti-inflammatory cytokines received a value of +1. If the food parameters were associated with reduced pro-inflammatory or increased anti-inflammatory cytokines, the article received a value of −1. Null values were set to 0. These scores were weighted based on study design. For example, randomized control trials received the greatest weight and cell culture the lowest weight. These scores and the weights were used to create pro- and anti-inflammatory fractions for each food parameter. The anti-inflammatory fraction was subtracted from the pro-inflammatory fraction to create the “article effect score” for each of the 45 food parameters. Additionally, DII calculation is linked to a regionally representative world database. The world database contains standard means and deviations for the 45 food parameters from 11 populations around the world (i.e., United States, United Kingdom, Bahrain, Mexico, Australia, South Korea, Taiwan, India, New Zealand, Japan, and Denmark) [12]. Dietary data in NHANES were collected using 24-h dietary recall interviews (24HR) conducted at the MEC [23]. The dietary interviews were administered by trained staff and the USDA’s Food Surveys Research Group was responsible for the dietary data collection methodology, maintenance of the databases used to code and process the data, and data review and processing [24]. 24HR-derived dietary information was used to calculate DII scores for all subjects, as described in detail elsewhere [12]. Higher (i.e., more positive) scores tend to indicate more pro-inflammatory diets and more negative values are more anti-inflammatory [12]. To control for the effect of total energy intake, the DII was calculated per 1000 cal of food consumed, which requires using the energy-standardized version of the world database.

### Statistical analysis

We conducted the analyses according to the Center for Diseases Control and Prevention guidelines for analysis of complex NHANES data, accounting for the masked variance and using the proposed weighting methodology [25–28]. We computed C-reactive protein, body-mass-index, smoking, race, age, education, and marital status-adjusted plasma TFAs level across quartiles of the DII by using analysis of covariance (ANCOVA) with Bonferroni correction. To determine any association between plasma TFAs and DII, we used multivariable-adjusted linear regression models adjusted for C-reactive protein, body-mass-index, smoking, race, age, education, marital status. All tests were two sided, and the level of significance as set at *p* < 0.05. We have applied complex sample analysis to deal with unequal probabilities of selection, nonresponse bias, and oversampling.

## Results

Of the 11,973 eligible participants, 47.8% (*n* = 5725) were men. The mean age was 47.1 years overall, 47.8 years in men and 46.5 years in women (*p* = 0.085). In all, 40.6% (*n* = 4864) of the participants had completed more than high school, 21.1% (*n* = 2522) had completed high school, while 30.6% (*n* = 3672) had completed less than high school. White (non-Hispanic) represented 45.1% (*n* = 5405) of the participants, Blacks 18.6% (*n* = 2230) and Mexican-Americans 23.2% (*n* = 2779). The distribution of marital status was 50.9% (5659) for married, 9.5% (*n* = 1055) for widowed and 9.3% (*n* = 1035) for divorced. Means and standard deviations for TFAs for subjects with information on TFAs concentration were 1.55 ± 0.52 μmol/L for *trans* 9-hexadecenoic acid, 3.18 ± 0.61 μmol/L for *trans* 11-octadecenoic acid, 2.96 ± 0.67 μmol/L for *trans* 9-octadecenoic acid and 0.68 ± 0.21 μmol/L for *trans* 9, *trans* 12-octadienoic acid, respectively. Mean BMI was 28.5 ± 6.6Kg/m^2^ overall, 28.0 ± 5.7 Kg/m^2^ in men and 29.0 ± 7.3 Kg/m^2^ in women (*p* = 0.012). DII score ranged from −5.33 to 4.24 with a median of −0.01 (25th–75th percentiles: −1.40-1.20).

Adjusted mean *Trans* 9-hexadecenoic acid, *trans* 11-octadecenoic acid, *trans* 9-octadecenoic acid, *trans* 9, and *trans* 12-octadienoic acid significantly increased across increasing quarters of the DII (*p* < 0.001, Table 1). In C-reactive protein, body-mass-index, smoking, race, age, education, and marital status adjusted linear regressions, *Trans* 9-hexadecenoic acid [β coefficient (β) 0.068 (95% CI: 0.032 to 0.188)], *trans* 11-octadecenoic acid [β 0.143 (95% CI: 0.155 to 0.310)], *trans* 9-octadecenoic acid [β 0.122 (95% CI: 0.120 to 0.277)], and *trans* 9, *trans* 12-octadienoic acid [β 0.103 (95% CI: 0.090 to 0.247)] were directly related to DII score (all *p* < 0.001).

**Table 1:** adjusted (C-reactive protein, body mass-index, smoking, race, age, education, marital status)mean of trans fatty acids across the quarters of the DII

Variables		Quarters of the dietary inflammatory index				*P*-value
		Quarter 1	Quarter 2	Quarter 3	Quarter 4	
Min and Max of DII		−5.33 to − 1.40	−1.40 to − 0.02	−0.02 to 1.20	1.20 to 4.24	
crude	*trans* 9-hexadecenoic acid	1.57 ± 0.03	1.63 ± 0.02	1.64 ± 0.03	1.56 ± 0.02	0.095
	*trans* 11-octadecenoic acid	3.10 ± 0.02	3.20 ± 0.02	3.29 ± 0.05	3.32 ± 0.07	<0.001
	*trans* 9-octadecenoic acid	2.92 ± 0.04	2.95 ± 0.02	3.02 ± 0.05	3.12 ± 0.03	<0.001
	*trans* 9, *trans* 12-octadienoic acid	0.65 ± 0.04	0.74 ± 0.02	0.76 ± 0.03	0.82 ± 0.02	<0.001
adjusted	*trans* 9-hexadecenoic acid	1.37 ± 0.04	1.40 ± 0.04	1.44 ± 0.02	1.50 ± 0.04	<0.001
	*trans* 11-octadecenoic acid	2.91 ± 0.03	2.96 ± 0.05	3.06 ± 0.04	3.32 ± 0.05	<0.001
	*trans* 9-octadecenoic acid	2.80 ± 0.04	2.82 ± 0.02	2.87 ± 0.03	2.96 ± 0.04	<0.001
	*trans* 9, *trans* 12-octadienoic acid	0.62 ± 0.04	0.69 ± 0.04	0.72 ± 0.03	0.80 ± 0.03	<0.001

Adjusted for C-reactive protein, body mass-index, smoking, race, age, education, marital status. Analysis of covariance applied. Value expressed as a mean SEM.DII, dietary inflammatory index

## Discussion

In this population-based sample, we have evaluated the association between plasma TFA and the DII. We found a positive association between plasma TFA and DII scores, which remained significant even after adjusting for a range the potential confounding factors.

Evidence from both observational and experimental studies indicates that TFAs are pro-inflammatory [5, 7, 29, 30], which is in line with our findings. The mechanisms underlying these effects are not well-established, but may involve TFAs incorporation into endothelial cell, monocyte/macrophage, or adipocyte cell membranes (affecting membrane signalling pathway relating to inflammation) or ligand-dependent effects on peroxisome-proliferator-activated receptor-g (PPAR-g) or retinoid X receptor (RXR) pathways [5, 7]. Activation of inflammatory responses and endothelial dysfunction may represent important mediating pathways between TFAs consumption and the risk of coronary heart diseases, sudden death, and T2DM [29]. It has been reported that TFA treatment of human aortic endothelial cell (HAEC) significantly increased the expression of endothelial adhesion molecules, including intercellular adhesion molecule-1 (CD54) and vitronectin receptor (CD51/CD61) [31]. Incorporation of TFA into membranes increased HAEC adhesion to fibronectin- or vitronectin-coated plates by 1.5- to 2-fold, respectively [31]. Neutrophil and monocyte adhesion to HAEC monolayers was nearly proportional to adhesion molecule expression. TFA treatment also induced the release of monocyte chemoattractant protein-1 by nearly threefold in non-stimulated HAEC [31]. TFA elevates inflammatory markers such as CRP, TNF alpha, and IL-6 [8, 32]. Mozaffarian et al. observed that TFA levels were positively associated with interleukin (IL) 1ß, IL-1 receptor antagonist, IL-10, tumor necrosis factor (TNF) α, TNF receptor 2, monocyte chemoattractant protein 1, and brain natriuretic peptide [7].

Some of the deleterious actions of the TFAs such as increasing insulin resistance have been attributed to its pro-inflammatory action. A recent study in mice demonstrated increased insulin resistance and signs of local inflammation in adipose tissue, as well as induction of cytokine gene expression after treatment with trans10cis12-CLA [33]. Adipocytes exposed to CLA secreted more pro-inflammatory cytokines than controls, and there was an increased macrophage infiltration in adipose tissue treated with trans10cis12-CLA [33]. Another mechanism, as indicated by many studies in CLA-fed mice, is down-regulation of PPAR-γ in adipose tissue [33–35], resulting in subsequent peripheral insulin resistance in skeletal muscle. Data in mice also suggest that CLA could promote a lipodystrophic state, with redistribution of fat stores, i.e. increased liver fat accumulation and decreased peripheral fat, also consistent with down-regulation of PPAR-g [35].

The effects of TFA consumption on cardiovascular events have not been evaluated in randomized controlled trials in humans. Such trials are unlikely to be performed, given the cost considerations, practicality, compliance and, perhaps most importantly, the ethical limitations of randomizing individuals to an intervention with strong evidence for significant harm. A prior meta-analysis of observational studies reported pooled relative risk estimates for CHD of 1.22 (95% confidence interval 1.08 to 1.38) for highest level of total intake of TFAs; 1.30 (0.80 to 2.14) for intake of industrially produced TFAs; and 0.93 (0.74 to 1.18) for intake of ruminant derived TFAs [36]. This suggests that industrially produced TFAs might increase the risk of CHD, though this also could reflect the low levels of ruminant derived TFAs compared with the higher doses of industrially produced TFAs typically consumed in studies and availability in the food supply [37]. A meta-analysis by Souza et al., reported that a 2% increase in energy intake from TFAs was associated with a 25% increased risk of CHD and 31% increase in CHD mortality [38]. Other meta-analyses also have reached the same conclusion [5, 39].

With regard to stroke, at least two prospective studies assessed the association between TFAs and ischemic stroke, and yielded inconsistent results [40, 41]. One study in men showed no association with stroke [40]; the other, in women, showed a positive association in those who did not take aspirin [41]. Furthermore, the association with TFAs was significant only for lacunar stroke, with a trend for haemorrhagic stroke, but not for stroke of cardioembolic origin. A nested case-control study conducted within the Women’s Health Initiative Observational Study with 10 year follow-up found no association between serum TFAs 16:1, 18:1, or 18:2 and ischemic stroke [42].

This study has several strengths. To our knowledge, it is the largest study of the association of plasma TFAs with the DII. The study is sufficiently powered to test the targeted associations. Participants were a random sample of the general population and therefore the results obtained from nationally representative samples can be extrapolated to the general population. As the data collection was performed on all days of the week throughout the year in NHANES, the potential for selection bias is very low [43, 44]. The findings from our study have to be considered in the context of some study limitations. The cross-sectional nature does not allow inference about causality; it is possible there is reverse causation, whereas it is not a case for our results since increased plasma TFAs cannot cause someone to eat a pro-inflammatory diet.

## Conclusions

Understanding the interplay between plasma TFA and DII is a necessary and important step toward any application of the resulting knowledge for public health policy and action. Our findings provide evidence on the association between plasma TFAs with the DII. The association of plasma TFAs and with a marker of inflammation suggests an underlying mechanism of harmful health effects.

## Abbreviations

ANCOVA: Analysis of covariance
CRP: C-reactive protein
CVD: Cardiovascular disease
DII: Dietary inflammatory index
E-DII: Energy-adjusted-dietary inflammatory index
HAEC: Human aortic endothelial cell
MEC: Mobile exam clinics
NCHS: National Center for Health Statistics
NHANES: National Health and Nutrition Examination Survey
PFB-Br: Pentafluorobenzylbromide
PPAR-g: Peroxisome-proliferator-activated receptor-g
RXR: Retinoid X receptor
T2DM: type 2 diabetes mellitus
TFA: *Trans* fatty acids
TNF: Tumour necrosis factor
